# Improving *in vitro* photodynamic therapy through the development of a novel iron chelating aminolaevulinic acid prodrug

**DOI:** 10.1016/j.pdpdt.2018.12.005

**Published:** 2019-03

**Authors:** Alison Curnow, Alexis Perry, Mark Wood

**Affiliations:** a European Centre for Environment and Human Health, University of Exeter Medical School, University of Exeter, Environment and Sustainability Institute, Penryn Campus, Cornwall, TR10 9FE, UK; b Biosciences, College of Life and Environmental Sciences, University of Exeter, Geoffrey Pope Building, Stocker Road, Exeter, Devon, EX4 4QD, UK

**Keywords:** Aminolaevulinic acid (ALA), AP2-18, Hydroxypyridinone (HPO), Iron chelation, Photodynamic therapy (PDT), Protoporphyrin IX (PpIX)

## Abstract

**Background:**

Photodynamic therapy (PDT) is a light activated drug therapy that can be used to treat a number of cancers and precancers. It is particularly useful in its topical form in dermatology but improvement of efficacy is required to widen its application.

**Methods:**

An ester between aminolaevulinic acid (ALA) and CP94 was synthesised (AP2-18) and experimentally evaluated to determine whether protoporphyrin IX (PpIX)-induced PDT effectiveness could be improved. A biological evaluation of AP2-18 was conducted in cultured human primary cells with both PpIX fluorescence and cell viability (as determined via the neutral red assay) being assessed in comparison to the PpIX prodrugs normally utilised in clinical practice (aminolaevulinic acid (ALA) or its methyl ester (MAL)) either administered alone or with the comparator iron chelator, CP94.

**Results:**

No significant dark toxicity was observed in human lung fibroblasts but AP2-18 significantly increased PpIX accumulation above and beyond that achieved with ALA or MAL administration +/- CP94 in both human dermal fibroblasts and epithelial squamous carcinoma cells. On light exposure, the combined hydroxypyridinone iron chelating ALA prodrug AP2-18 generated significantly greater cytotoxicity than any of the other treatment parameters investigated when the lowest concentration (250 μM) was employed.

**Conclusions:**

Newly synthesised AP2-18 is therefore concluded to be an efficacious prodrug for PpIX-induced PDT in these dermatologically relevant human cells, achieving enhanced effects at lower concentrations than currently possible with existing pharmaceuticals.

## Highlights

• A new combined iron chelating prodrug (AP2-18) has been synthesised and evaluated.
• AP2-18 significantly increased protoporphyrin IX accumulation in human skin cells.
• This enhancement translated into greater cytotoxicity on irradiation.
• Clinical AP2-18 application may improve future dermatological photodynamic therapy.

## Graphical abstract

**Figure:**
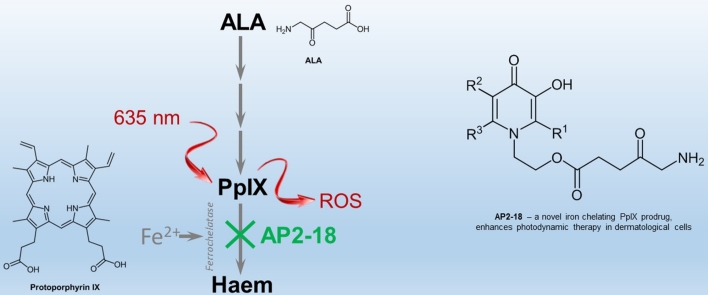

## 1 Introduction

Non-melanoma skin cancer (NMSC) is the most common form of cancer worldwide [1] and its causation is predominantly associated with excessive solar ultraviolet radiation exposure [2]. NMSC prevalence is increasing, with over 80,000 new cases reported in England in 2008 and NHS skin cancer costs predicted to approach £180 M by 2020 [3]. Efficacious, cost-effective and cosmetically acceptable NMSC treatments are therefore essential.

Traditional treatment of NMSC commonly includes surgical excision, topical 5-fluorouracil or cryotherapy [[4], [5], [6], [7]]. However, these conventional therapies are not always associated with excellent cosmesis [[4], [5], [6], [7]] and their appropriateness can be limited, depending on the location, size and number of NMSC lesions to be treated [[7], [8], [9]]. Photodynamic therapy (PDT) is a relatively new light-induced drug treatment for certain types of NMSC that is minimally invasive [[4], [5], [6], [7], [8], [9], [10]]. Delivery of dermatological PDT can be nurse-led and is safe, with few side effects beyond treatment effects [5,6,8,9,11]. Several cancers, including NMSCs and pre-cancers can be treated using PDT [[4], [5], [6], [7], [8], [9], [10], [11], [12], [13]] as well as some non-malignant skin diseases such as psoriasis and acne [14,15].

PDT uses light to activate a pre-administered drug in the presence of molecular oxygen to kill diseased, precancerous or neoplastic cells without harming healthy cells, so healing occurs without scarring [15]. It also has several advantages over some forms of traditional management, including the possibility of treating a whole area of field change at once, the occurrence of good healing on the lower leg, repeated application to the same area of the skin without the development of resistance, excellent cosmetic results in highly visible sites without the need for advanced surgical techniques and its compatibility as an adjuvant with other treatment approaches [[4], [5], [6], [7], [8], [9], [10], [11], [12], [13], [14]].

The photosensitiser most commonly used in dermatological PDT is protoporphyrin IX (PpIX) [8,9,12]. PpIX (a large, water-insoluble molecule) can be excited by light of wavelength 635 nm [16]. Skin lesions are therefore treated with a topical cream containing a small, soluble precursor to PpIX (e.g. 5-aminolaevulinic acid (ALA) or the methyl or hexyl-esters of ALA, methyl-aminolevulinate (MAL) or hexyl-aminolevulinate (HAL) respectively) [8,9]. These precursors are absorbed by cells and enzymatically converted into light sensitive PpIX over a few hours (typically three in clinical application) by the haem biosynthesis pathway naturally present in all nucleated cells [8,16,17]. This exogenous administration of copious amounts of PpIX precursor bypasses the primary rate limiting step of this pathway (the synthesis of ALA from glycine and succinyl-CoA by ALA synthase) [[16], [17], [18]]. This forces the rest of the pathway to operate at maximal capacity until PpIX (the immediate precursor to haem) is formed. This naturally light sensitive compound starts to accumulate over time as the final step in the pathway (the insertion of Fe^2+^ into PpIX by ferrochelatase to form haem) is relatively slow to occur and is thus the secondary rate limiting step of this pathway [[16], [17], [18]]. It is important to note that haem biosynthesis is elevated and less well controlled in cancer cells, which also possess an altered iron metabolism and dysregulated porphyrin biosynthesis enzymes. This makes neoplastic cells more prone to accumulate PpIX more rapidly than normal cells and thus results in this treatment approach being relatively selective [16,19,20]. The disrupted tumour surface is also more permeable than healthy skin to the topical application of the prodrug cream, facilitating PpIX precursor penetration to where its treatment action is needed most [16,20].

Although effective treatment outcomes associated with excellent cosmesis have been demonstrated with PpIX-induced PDT conducted in dermatological lesions where this treatment modality is licensed (actinic keratosis, Bowen’s disease and BCC) and when the disease remains superficial [8,21], efforts continue to both increase the efficacy and extend the applications of dermatological PDT particularly in order to treat thicker or acrally located conditions [22]. It is already known that poor penetration into the deeper skin layers can be improved clinically by employing more lipophilic ALA derivatives (e.g. MAL; Metvix, Galderma, UK) [[23], [24], [25], [26], [27]] or nanoemulsion formulations (e.g. ALA; Ameluz, Spirit Healthcare, UK) [21] and by performing skin pre-treatments like the removal of the outer skin layer by tape stripping or scraping [[28], [29], [30]]. In fact many adaptations to the standard treatment protocol have been considered to improve efficacy including skin pre-treatment with the malignant cell differentiation potentiator dimethyl sulfoxide [31], light dose fractionation [32,33], low fluence rate light administration [34] as well as combinations with other techniques (e.g. low dose Photofrin [35], hyperthermia [36,37], iontophoresis [38] and bioreductive drugs [39].

**Scheme 1:**
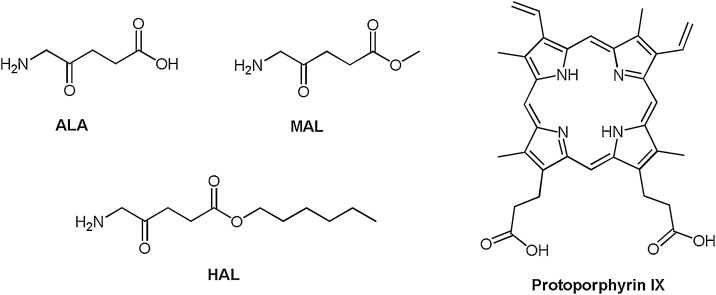
The molecular structures of the active photosensitiser protoporphyrin IX (PpIX) and its existing prodrugs (aminolaevulinic acid (ALA) and its methyl (MAL) and hexyl esters (HAL)).

**Scheme 2:**
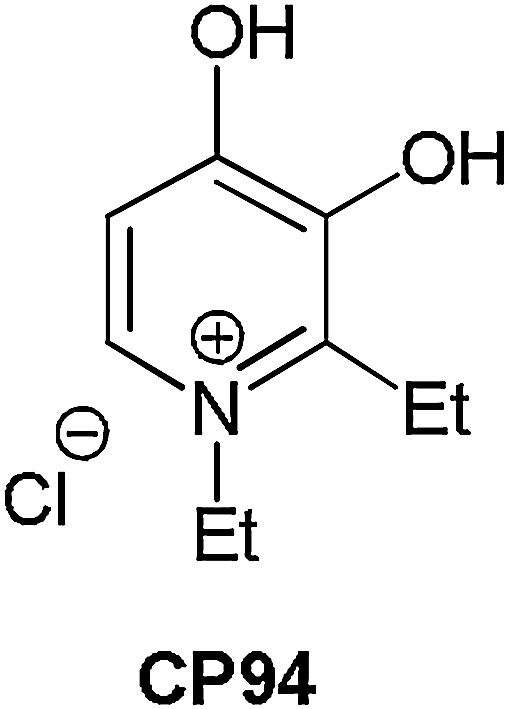
The molecular structure of the existing hydroxypyridinone iron chelating agent (represented here as its pyridinium salt tautomer), CP94 (1,2-diethyl-3-hydroxypyridin-4-one).

A simple pharmacological solution however, utilises the administration of an iron chelating agent to temporarily inhibit the final stage of the haem biosynthesis pathway, which requires iron to convert PpIX into haem [40]. This pathway is required to convert each of the commercially available PpIX prodrugs (ALA, MAL and HAL) into the active photosensitiser PpIX [41] (Scheme 1). Designing a non-toxic, topically available iron chelator is technically demanding and not all clinically available iron chelators are suitable for a single topical and concomitant administration with PpIX-PDT. CP94 (1,2-diethyl-3-hydroxypyridin-4-one), is a hydroxypyridinone iron chelating agent, which has been found however, to have all the parameters required for this purpose (Scheme 2).

Direct comparison of CP94 and desferrioxamine (DFO; Desferal; an established iron chelator administered clinically by long infusion for the treatment of iron overload) in cultured human lung fibroblasts and epidermal carcinoma cells has previously established [42] that CP94 is a better enhancer of ALA/MAL-induced PpIX fluorescence than DFO (and it is already known that DFO is a better enhancer of PpIX-PDT than EDTA [40]), probably as a result of its lower molecular weight, greater lipophilicity and neutral charge enabling it to access intracellular iron pools more rapidly [43]. Further *in vitro* experimentation has also indicated that CP94 is also a better enhancer of ALA/MAL/HAL-PDT than the already clinically established iron chelator dexrazoxane (a bisdioxopiperazine iron chelating agent similar to EDTA, which can administered clinically as an injection alongside doxorubicin to act as a cardioprotective agent in women receiving treatment for metastatic breast cancer) [44] and works in a number of different human cell types [45].

Furthermore, it has been established *in vivo* in a rat colon model that CP94 can be used to significantly enhance PpIX levels [46]. Additionally on irradiation in this model, CP94 + AlA has been found to produce three times the area of necrosis produced by ALA alone when delivered at the same time point [46]. Subsequent studies in a rat colonic tumour model determined that iron chelation with CP94 was just as effective at enhancing ALA-PDT as light dose fractionation when using comparable parameters [47]. A topical preparation was also derived and was found to be effective at enhancing ALA-PDT in rat skin [48]. Other investigations of CP94 as an enhancer of PDT have taken place in rat bladder [49] and rabbit uterus [50]. In addition, the iron chelating capacity and toxicity of CP94 have been investigated in a number of iron overloaded and non-overloaded animal models including mice [51,52], rats [53,54], guinea pigs [54], rabbits [55] and Cebus monkeys [53]. Rat studies were found to be predictive of the iron chelating capacity recorded in primates [53] and the metabolism of CP94 in guinea pigs was noted to be more similar to that of humans [54] than studies undertaken in rats as this compound is glucuronidated by the liver and excreted in urine.

Following these promising experimental findings, topical CP94 administration has been investigated in humans as way of improving PpIX-induced PDT. Dose escalating pilot studies in nodular basal cell carcinoma (one treatment cycle without lesion debulking) with simultaneous topical ALA [56] or MAL administration [11] have already been conducted. In both cases, CP94 administration was found to be a safe, effective and feasible treatment modification which did not produce any additional adverse reactions (no skin irritation during cream application, no increased erythema following cream application, no increase in pain visual analogue scores during irradiation and liver function assessed by blood tests remained unaltered in all patients). Histological analysis also indicated a significant, increased trend towards complete clearance with increased concentrations of CP94 (up to 40% w/w). It should be noted that efficacy would have been much improved if normal lesion pre-treatment had been conducted but it was decided not to do this in these initial clinical studies, so that histological measurements could be employed as endpoints. CP94 [57] and its close relation relation, CP20 (1,2-dimethyl-3-hydroxypyridin-4-one; L1; Deferiprone; DFP) have also been investigated as iron chelating agents in humans not undergoing PDT, with the latter being studied in detail in long term investigations of daily oral administration [58,59]. There is rapid first pass metabolism by the liver following oral administration of CP20 in humans [60] which limits the clinical effectiveness of these particular hydroxypyridinone iron chelators for the treatment of conditions of iron overload [61,62], resulting in Deferasirox administration being utilised, either on its own or in combination with the established Deferoxamine long infusion treatment regime [63]. These issues are avoided however, when the iron chelating agent is applied on single occasions topically to a localised treatment area on the skin during PpIX-PDT and the subsequent rapid metabolism actually then becomes an advantage in this application as its effects are short-lived, minimising any potential systemic toxicity.

Effective as it is, as CP94 and its effect on PpIX-induced PDT is already in the public domain, it therefore cannot be protected substantially enough to make it a commercially attractive pharmaceutical. A novel combinational prodrug has therefore been developed, synthesised [64] and tested specially for this purpose here. AP2-18 contains both the iron chelating capacity of CP94 as well as a PpIX-inducing prodrug, ALA.

## 2 Materials and methods

### 2.1 AP2-18 synthesis

**Scheme 3:**
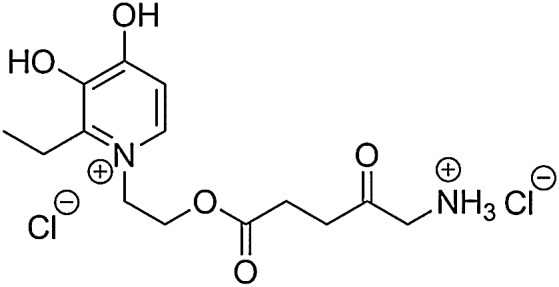
The molecular structure of the new hydroxypyridinone iron chelating ALA prodrug, AP2-18.

In order to produce a novel pharmaceutical compound for use in photodynamic therapy (PDT) the iron chelating compound 1,2-diethyl-3-hydroxypyridin-4-one hydrochloride (CP94) was coupled to protoporphyrin IX pro-drug aminolaevulinic acid (ALA) via an ester linkage to produce the iron chelating pharmaceutical compound AP2-18 (C_14_H_22_Cl_2_N_2_O_5_; Scheme 3). We filed a patent application describing the synthesis of AP2-18 in 2012 and this has been subsequently granted following scrutiny by numerous individual countries and international territories [64].

### 2.2 Cell culture

All media and disposable plastic equipment were purchased from Sigma (Poole, UK) unless otherwise stated. Human fetal lung fibroblasts (MRC-5), human skin fibroblasts (84BR) and human epidermal skin carcinoma cells (A431) were obtained from the European Collection of Cell Cultures (ECACC, Wiltshire, UK). Cells were maintained in Eagle’s Minimum Essential Medium (EMEM) supplemented with 10% fetal calf serum (20% for 84BR cells), 2% (200 mM) l-glutamine and 2% (200 U/ml) penicillin and (200 μg/ml) streptomycin solution. Cells were incubated at 37 °C and 5% CO_2_ and passaged every 3–5 days as required. All cell culture work was carried out using aseptic technique in a Class II biological safety cabinet.

### 2.3 Initial dark toxicity testing

**Fig. 1:**
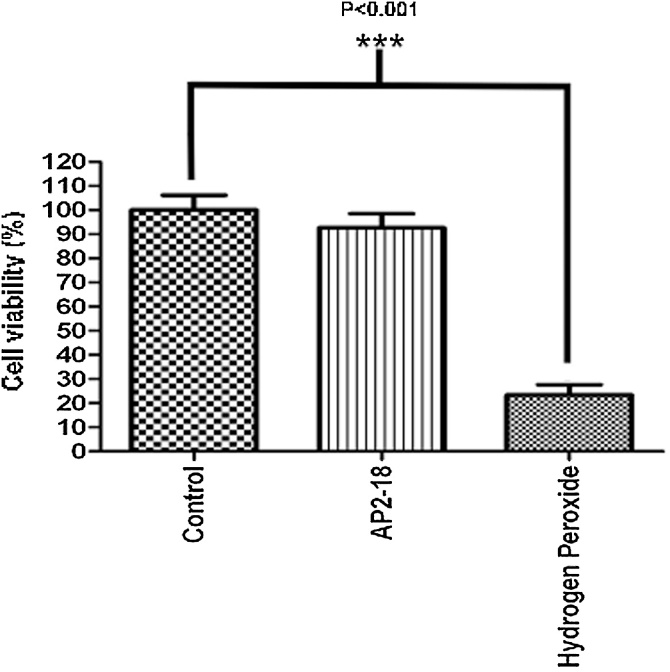
Neutral red uptake assay to assess the level of inherent (dark) toxicity possessed by AP2-18. AP2-18 was not found to be inherently toxic to human lung fibroblasts (MRC-5) when compared to control cells only exposed to cell medium. Exposure to the 0.01% (v/v) hydrogen peroxide positive control conditions resulted in a significant (P < 0.001; Student’s t-test) reduction in cell viability.

To establish if the compound AP2-18 possessed any inherent toxic properties prior to experimentation, a 1000 μM test solution was prepared (the highest concentration used during this investigation) in standard cell culture medium without phenol red (colourless EMEM). This was applied to MRC-5 cells (human lung fibroblasts; pre-seeded into 96 well plates at 5 × 10^4^ cells per well and incubated for 24 h in 200 μl of media to obtain 80% confluency), under reduced light conditions and left for 4 h in the dark. Concurrently, further cells were also exposed to 0.01% (v/v) hydrogen peroxide, which acted as a positive control or to cell media alone, as a blank control. Cell viability was then determined using the neutral red uptake (NRU) assay (Fig. 1).

Neutral red is an inert dye actively taken up and stored by viable (living) cells, an action which is unable to be performed by non-viable cells, therefore the level of neutral red taken up is directly proportional to the number of viable cells present following a given exposure [65,66]. Briefly, the test solutions were aspirated and the cells washed with 200 μl of 0.3% Bovine Serum Albumin (BSA) in PBS. 100 μl of 0.05% neutral red in PBS was then added to each well for 8 min at room temperature before three more washes (each with 200 μl 0.3% BSA in PBS) were conducted and 100 μl 70% ethanol containing 0.37% HCl was applied. Absorbance at 534 nm was then measured with a plate reader (Synergy HT, BIO-TEK, Germany) with the absorbance at 405 nm being subtracted to determine the number of cells that survived the PDT treatment.

### 2.4 PpIX fluorescence accumulation

The level of PpIX (the light sensitive photosensitiser produced from the prodrugs administered in this form of PDT) accumulation was monitored using a well-established, previously validated, fluorescence based assay employed within our laboratory [11,43,44,67]. Briefly, cells were seeded at 2 × 10^4^ cells per well in a 96 well plate and left to adhere overnight. Test solutions were prepared on the day of the assay by dissolving ALA, MAL, CP94 or AP2-18 into colourless EMEM (i.e. minus phenol red). The pH of the solutions were checked and adjusted to physiological pH (pH 7.4) using NaOH (0.5 M) as necessary. Solutions were then filter sterilised (0.22 μm, Millipore filter) before being diluted to the final concentrations and applied to the cells. CP94 (1,2-diethyl-3-hydroxypyridin-4-one hydrochloride) was kindly provided as a powder by Professor Hider (King’s College London, UK).

The level of PpIX produced was monitored using a multi-well fluorescent plate reader (Synergy HT, BIO-TEK, Germany) with a 400 (± 30) nm excitation filter and a 645 (± 40) nm emission filter, with the level of fluorescence produced being directly proportional to the level of PpIX present. Readings were taken hourly for 6 h and were conducted under low light conditions to reduce any photobleaching of PpIX from occurring.

**Fig. 2:**
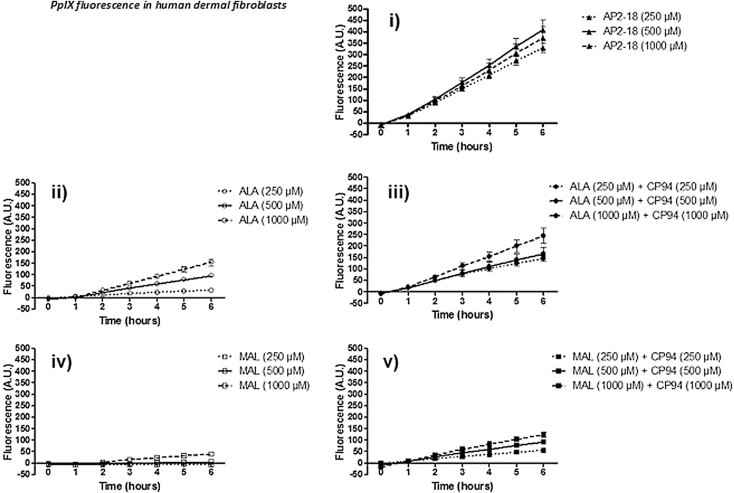
Accumulation of PpIX fluorescence (+/- the standard error of the mean) in human dermal fibroblasts (84BR) over time following exposure to i) novel compound AP2-18, ii) ALA alone, iii) ALA + the iron chelator CP94, iv) MAL alone and v) MAL + the iron chelator CP94. Statistical comparison provided in S1.

**Fig. 3:**
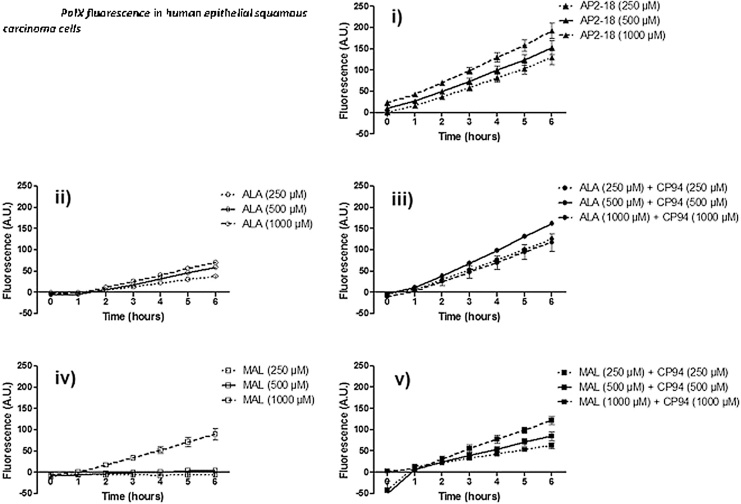
Accumulation of PpIX fluorescence (+/- the standard error of the mean) in human epithelial squamous carcinoma cells (A431) over time following exposure to i) novel compound AP2-18, ii) ALA alone, iii) ALA + the iron chelator CP94, iv) MAL alone and v) MAL + the iron chelator CP94. Statistical comparison provided in S2.

To evaluate the ability of AP2-18 to cause PpIX accumulation within cells, a series of concentrations were prepared (250 μM; 500 μM; 1000 μM), which reflect the range previously employed by our group during similar experimentation with PpIX precursors and iron chelating agents [11,43,44,67]. These were tested alongside equimolar concentrations of ALA and CP94 (the components of AP2-18) and methyl aminolevulinate (MAL) (the licenced prodrug currently widely employed in dermatological PDT in the UK), with all test compounds being investigated in both human dermal fibroblasts (84BR; Fig. 2) and human epithelial squamous cell carcinoma cells (A431; Fig. 3).

The results of the statistical analyses which were subsequently undertaken are presented in S1 and S2 (84BR and A431 cells respectively).

### 2.5 PDT efficacy

**Fig. 4:**
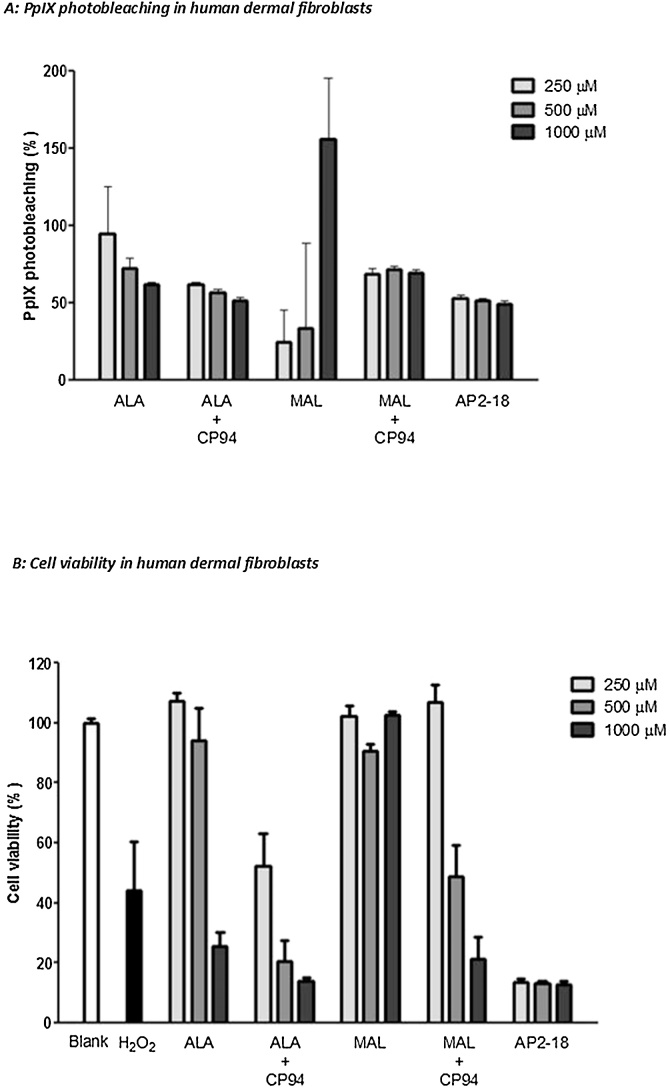
Percentage PpIX photobleaching (A) and effect on viability (B) of human dermal fibroblasts (84BR) following exposure to AP2-18, ALA +/- CP94 or MAL +/-CP94 and irradiation with red light. Statistical comparison provided in S3.

**Fig. 5:**
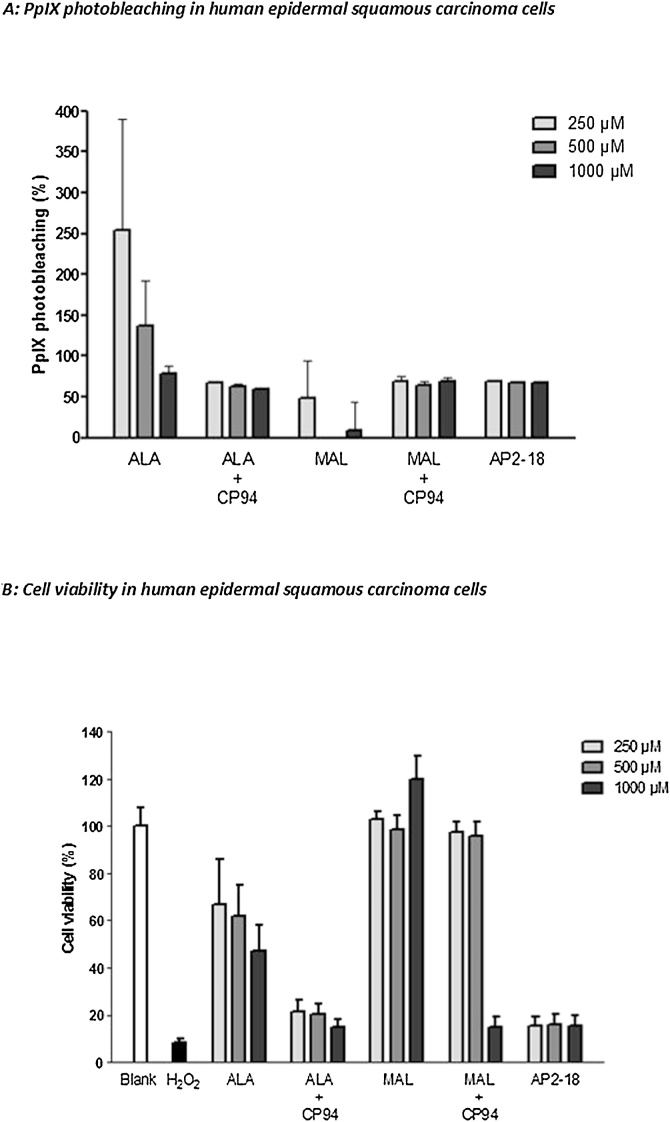
Percentage PpIX photobleaching (A) and effect on viability (B) of human epithelial squamous carcinoma cells (A431) following exposure to AP2-18, ALA +/- CP94 or MAL +/-CP94 and irradiation with red light. Statistical comparison provided in S4.

To assess the effect of AP2-18 on PpIX- induced PDT efficacy, the same dermatologically relevant cells were exposed to equimolar concentrations of AP2-18, ALA +/- CP94 or MAL +/- CP94 (as described above) and incubated in the dark for 4 h as this time point yielded promising results in the fluorescence studies described above. The level of PpIX accumulation was then quantified as before, prior to irradiation with red light (37 J/cm^2^; 635 ± 2 nm; Aktilite, Galderma, UK) as well as immediately afterwards so that the change in PpIX level (PpIX photobleaching) could be calculated as a percentage (Fig. 4A and 5A  with dermal fibroblasts and squamous carcinoma cells respectively). Cell viability was then assessed using the NRU assay (as described above) with these data being normalised against the blank control cells (which were exposed to normal cell media) and presented as a percentage of viable cells (dermal fibroblasts Fig. 4B and squamous carcinoma cells Fig. 5B). The results of the statistical analyses which were subsequently undertaken are presented in S3 and S4 (84BR and A431 cells respectively). These data presented are the mean of three independent experiments each consisting of three internal repeats of each condition.

## 3 Results

Treatment with AP2-18 did appear to result in a very slight reduction in the number of viable MRC5 cells (Fig. 1), however on statistical analysis this was not found to be significantly different to control cells incubated in standard cell culture medium. It was therefore concluded that AP2-18 does not possess any inherent toxicity and could be tested for efficacy in dermatologically pertinent cells.

Accumulation of PpIX fluorescence produced by each of the prodrugs investigated (AP2-18, ALA +/- CP94 or MAL +/- CP94) increased over time in each cell type examined; human dermal fibroblasts (Fig. 2) and human epidermal squamous carcinoma cells (Fig. 3). AP2-18 was also found to significantly increase PpIX accumulation in both dermatological cell types, above and beyond that achieved with ALA or MAL administration either alone or in combination with the comparator iron chelating agent, CP94. This enhancement was most consistent at incubation times of 4 h and greater in the dermal fibroblast cell type. ALA was also observed to be more effective at producing PpIX, than the same concentration of MAL administered in both the cell types investigated. Additionally higher levels of PpIX fluorescence were generally recorded in the dermal fibroblasts than the squamous carcinoma cells. As these PpIX fluorescence studies indicated that AP2-18 had the potential to substantially improve PpIX-induced PDT, further experimentation was undertaken to determine whether these significant increases in PpIX accumulation could be translated into increased cell kill on irradiation.

Substantial PpIX photobleaching (i.e. a reduction in PpIX fluorescence during light irradiation) was observed in the vast majority of the treatment groups investigated (Figs. 4A and 5 A). This demonstrated that PpIX was being “consumed” during the light treatment through an unwanted reaction (as PpIX was being transformed into a non-PDT active photoproduct through interaction with singlet oxygen being produced by the PDT reaction itself) and thus indicated that PDT was successfully occurring within both cell types investigated. Complete PpIX photobleaching was rarely achieved with the particular treatment parameters employed here however and this may indicate increased PDT effects could have been delivered if a bigger light dose had been employed. However, with the exception of the MAL only treatment groups, there was a general trend of greater photobleaching being observed with increasing doses of the PpIX precursors being administered.

Analysis of the cell viability results revealed that both the blank control and hydrogen peroxide positive control groups performed as anticipated in both cell types, producing little cytotoxicity and considerable cell death respectively. In human dermal fibroblasts (Fig. 4B) the use of the iron chelator CP94, improved the PDT effect of both ALA and MAL in a concentration dependent manner and AP2-18 was found to be significantly better than all the MAL doses and all the ALA doses except the highest (1000 μM) considered, at reducing cell viability following PDT even when the lowest concentration of AP2-18 (250 μM) was employed (S3). At 1000 μM ALA and with the higher doses of CP94 administered in combination with ALA or MAL, when statistical significance was not achieved, the level of cell kill produced by AP2-18 was equivalent to that observed with the other treatment groups. These neutral red viability assay results also appeared to correlate with the PpIX fluorescence and photobleaching results noted above, with ALA again appearing more effective than MAL in terms of cell kill in this cell type. Very similar trends and significant reductions in cell viability were also observed in the human epithelial squamous carcinoma cells (Figs. 5B & S4), where once again AP2-18 and ALA + CP94 were the most effective PDT protocols tested producing not statistically dissimilar results from one another but substantially better effects than the ALA or MAL congeners administered alone. The addition of the hydroxypyridinone iron chelating agent CP94, either separately to ALA administration or via the new combinational iron chelating ALA prodrug AP2-18, therefore significantly improved PpIX-PDT in these human skin cells.

## 4 Discussion

AP2-18 has therefore been found to significantly increase PpIX accumulation in both human dermal fibroblasts (84BR) and human epithelial squamous carcinoma cells (A431) above and beyond that achieved with ALA or MAL administration either alone or in combination with the iron chelator CP94. Furthermore it has also been found that AP2-18 produced significantly increased PDT cell kill on irradiation and achieved this effect at lower concentrations than possible with ALA or MAL with or without administration of the iron chelator CP94 in these cells of dermatological origin. In the squamous carcinoma cells, AP2-18 was demonstrated to be equally effective as ALA + CP94 administration, demonstrating the biological effectiveness of the newly synthesised compound.

AP2-18 has therefore been demonstrated *in vitro* to be an efficacious and improved prodrug for PpIX-induced PDT, which warrants further detailed study. Although we know the toxicity profile, metabolism route and efficacy of all the active components of AP2-18 (ALA, MAL, HAL, PpIX and CP94) in a variety of animal models as well as human beings, a portion of this work (i.e. key experiments and doses) will need to be repeated with the new combinational prodrug AP2-18 to i) ensure future patient safety, ii) gain regulatory approval and iii) determine the most effective treatment protocol for clinical application.

These results indicate that AP2-18 is able to enter human cells and additionally, because increased PpIX production results, it can be hypothesised that cytosolic esterases are able to break the bond between the PpIX prodrug and CP94 molecule, so that these constituent parts can function effectively. Once split the potential toxicity profile and metabolism route of the compounds should be as previously observed. Experimentation will have to be undertaken in animals however, to confirm this when using the new combination prodrug AP2-18 is utilised as the starting point. Additionally, topical availability and PDT effectiveness will have to be confirmed in suitable animal models and although theoretically this will not be an issue, it will still need to be demonstrated through future research before clinical evaluation can be considered. The topical availability of AP2-18 would probably be best evaluated in an animal model such as pig skin prior to conducting clinical studies. These evaluations could mirror the successful approach adopted by Maisch et al. (2010), when they evaluated PpIX distribution of a topically applied nanoemulsion-based formulation of BF-200 ALA in a porcine *ex vivo* skin model in comparison to MAL [68]. The amount of work required to do all this will be significantly less than with other agents at this stage of development however, because of the extensive research and clinical experience already gained with the active components of this new combinational prodrug [11,[42], [43], [44], [45], [46], [47], [48], [49], [50], [51], [52], [53], [54], [55], [56], [57],^67^,^69^,^70^].

AP2-18 is effectively a combination of ALA and the hydroxypyridinone iron chelating compound CP94, which have been linked via an ester linkage. However, our biological evaluation in these dermatologically derived cell types, indicate that this linked compound appears to have a similar or better activity profile than a PpIX precursor administered separately in combination with CP94 and at lower doses in many cases. It might have been reasonably expected that delivering ALA and CP94 in a linked format (rather than separately) may have altered the way the compounds enter cells in potentially a negative manner; bigger molecules tend to not enter cells as effectively as smaller molecules and may use different transporters. In fact, even the molecularly very similar structures of ALA and MAL prodrugs are thought to enter cells via different membrane transporters in different cell types but in the A431 squamous carcinoma cells employed here, cell uptake of both ALA and MAL is thought to be via Gat-3 (GABA) transporters [71]. The cellular uptake mechanism of AP2-18 has not been investigated here but there was no guarantee that the new entity would be able to produce even the same level of results as ALA and CP94 administered as separate agents and so these *in vitro* findings are encouraging. These findings are also supported to some extent by the work of Battah et al. [72], who made AP2-18 (compound 7a [72]) as part of a large series of hydroxypyridinone and 5-aminolaevulinic acid (HPO-ALA) conjugates that they have compared with ALA activity, subsequent to the publication of our patent [64]. Although this published experimentation cannot be directly compared to the results presented here due to methodological differences such as different cell lines, blue light and physiological levels of iron supplementation being employed, they concluded that passive diffusion appeared to be the main mechanism of cellular entry for the majority of the HPO-ALA conjugates that they studied and that ester binding the hydroxypyridinone to ALA via longer hydrocarbon linkages of up to n = 10 produced greater PpIX fluorescence over the same incubation time period of 6 h. Furthermore, these observations translated into increased phototoxicity being detected with the MTT assay on illumination and no appreciable HPO-ALA dark toxicity was observed [72]. There is therefore increasing experimental evidence that HPO-ALA conjugates may be a very useful way of enhancing the efficacy of the clinical application of PpIX-PDT.

It is actually very difficult to predict how the linked format of PpIX precursor to an iron chelating hydroxypyridinone might affect the innate cellular biochemistry relied upon to produce the natural photosensitiser PpIX. ALA is normally formed by ALA synthase in the mitochondrion before entering the portion of the haem biosynthesis pathway that occurs in the cytosol. The later step of insertion of iron into the PpIX porphyrin ring to form haem occurs in the mitochondrion [16]. In order to influence this pathway in such a way that PpIX accumulates, the iron chelator needs to be able to diminish mitochondrial levels of iron either directly or indirectly. However, AP2-18 first needs to be separated into its active components by esterases present in the cytosol in a similar manner to MAL [73] and Battah et al. hypothesise that a longer hydrocarbon linkage improves the cellular uptake of HPO-ALA conjugates by making them less hydrophilic [72], however generally smaller molecules enter the skin more easily. In addition, in theory it might seem better to deliver the CP94 before the ALA, in order to chelate the iron prior to producing the PpIX, whereas delivering the agents in a linked format means that the agents are delivered simultaneously. However, it appears quite the opposite is true both here with the new AP2-18 combined structure and in previous work in animals with ALA and CP94 administration undertaken at different time points [46].

Furthermore, the iron chelator CP94 is bidentate and it therefore takes three CP94 molecules to bind one Fe^2+^ ion. In addition to this, in the haem biosynthesis pathway two molecules of ALA dimerize to form porphobilinogen after which four molecules of the latter are condensed, rearranged and cyclised to produce uroporphyrinogen III; this is then converted into protoporphyrin IX via coproporphyrinogen III. Therefore, eight molecules of ALA are needed to form one PpIX molecule, which binds to one Fe^2+^ ion to form one molecule of haem [16]. The theoretical ratio of ALA : CP94 required per Fe^2+^ ion would, therefore, in simplistic biosynthetic terms, be 8 ALA : 3 CP94, i.e. over twice as much ALA as CP94. Despite this, the equal quantities of ALA and CP94 delivered via the combined AP2-18 precursor compound appeared highly effective in the cell types investigated here and could theoretically be due to greater levels of CP94 delivered via AP2-18 administration, draining intracellular iron stores, making haem formation from PpIX less likely to occur and thus augmenting PpIX levels as well as leading to greater PDT effects on irradiation as observed here.

In our previous investigations of ALA and CP94 co-administration [698], where we controlled the amounts of iron supplied to the cells in the tissue culture media, we found that iron chelation had a highly positive effect on PpIX accumulation, which far outweighed any limiting factor that reduced iron availability might have on subsequent free radical production on irradiation, and this led to enhanced PDT effects being produced following the administration of an iron chelating agent. It is clear however, that we are manipulating a highly complex biochemical environment, which can vary depending on cell type and innate iron status.

It is therefore concluded that a novel, combined hydroxypyridinone iron chelating ALA prodrug (AP2-18) has been synthesised and demonstrated to be more effective at generating PDT effects when investigated *in vitro* in human dermatological cells, than existing clinically utilised PpIX prodrugs. With further investigation, the significant increases in cytotoxicity following PpIX-induced PDT conducted with AP2-18, could potentially be translated into clinical PDT settings to produce substantial benefits for patients. This could either take the form of reducing the number of treatment cycles currently required to treat existing licensed indications or alternatively improve outcomes in dermatological applications where a more effective PDT treatment regime is still required.

## Funding sources

This work was supported by a Proximity to Discovery grant from the Medical Research Council, UK and a research grant from Killing Cancer, UK.

## Competing interest statement

A.Curnow, M Wood and A Perry have patent PCT/GB2013/052297 issued.

## Figures and Tables

**Figure:**
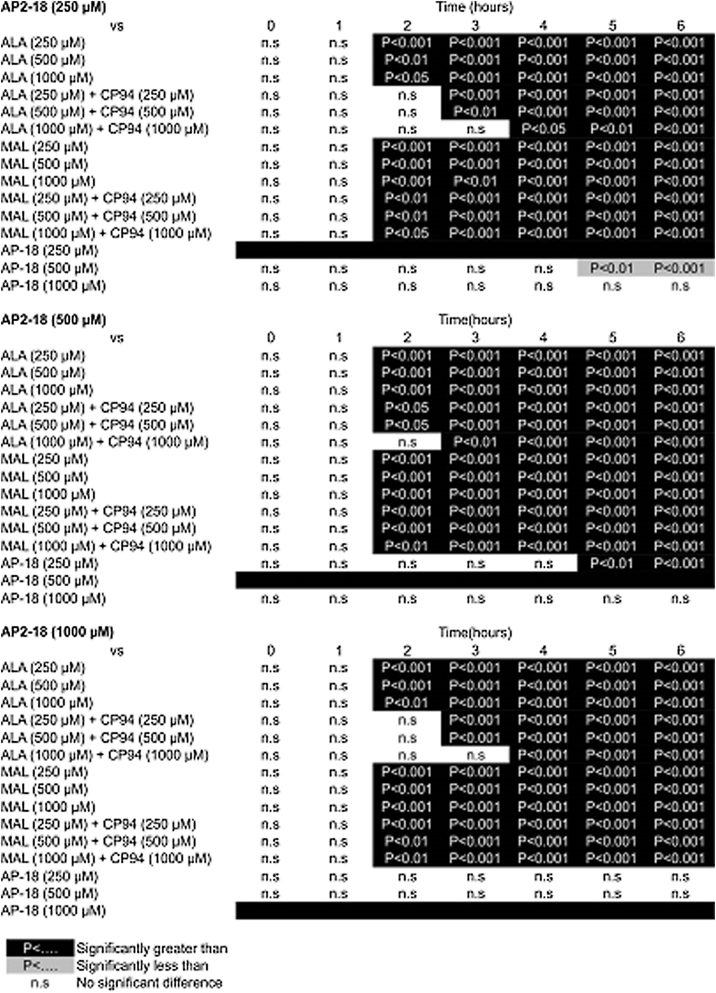


**Figure:**
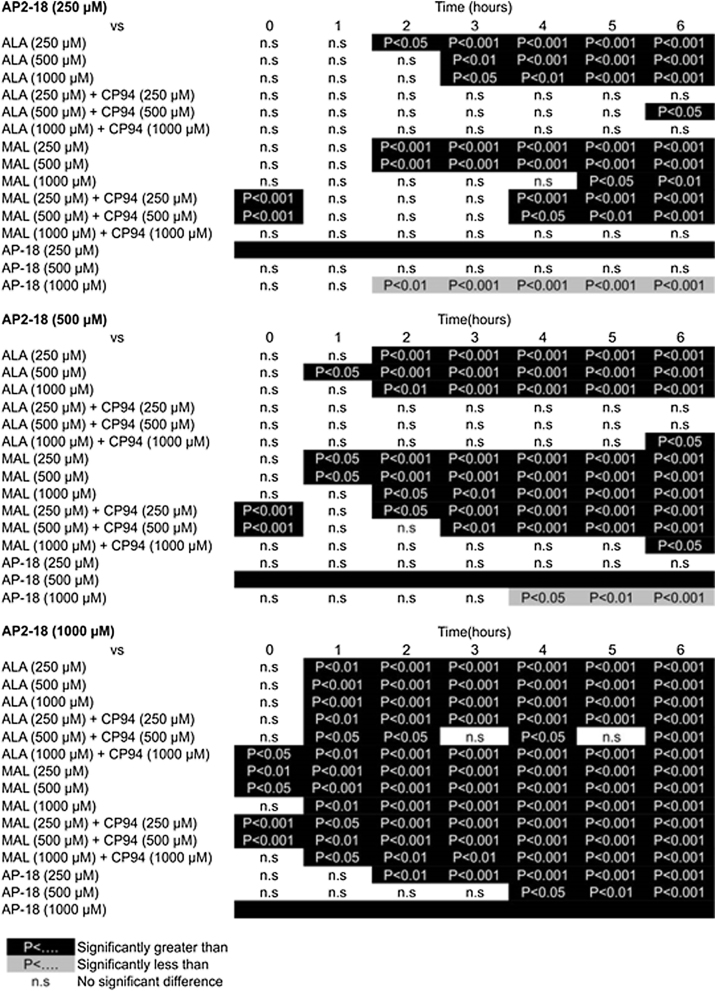


**Figure:**
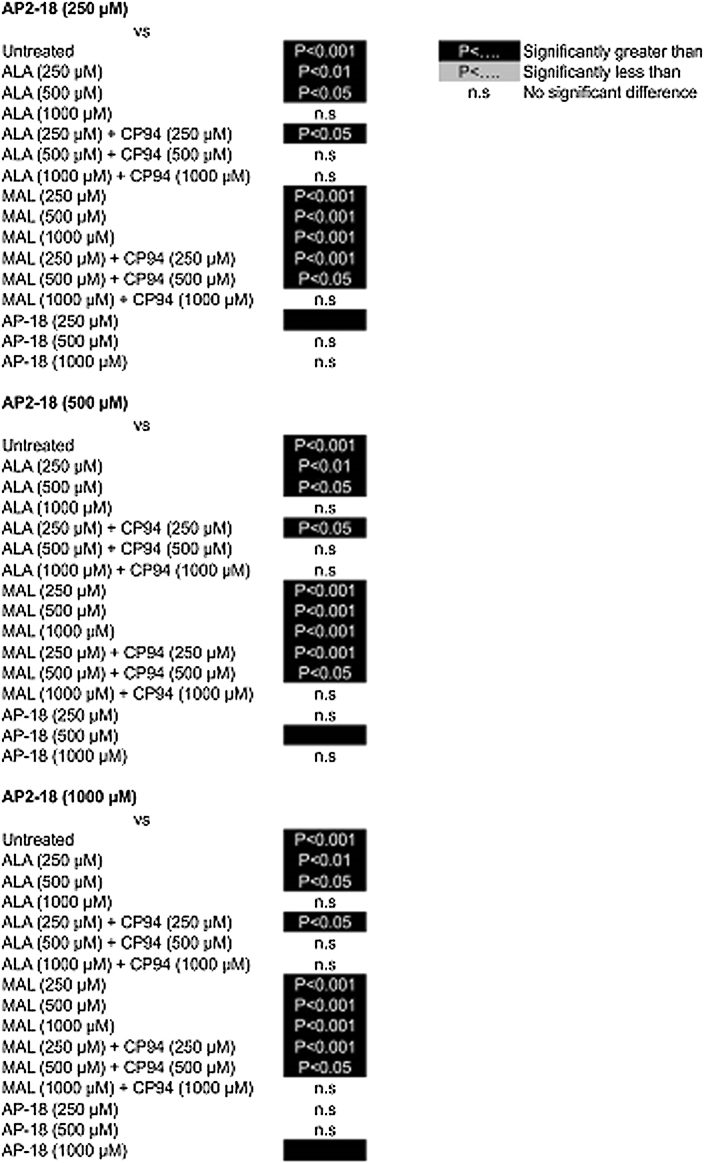


**Figure:**
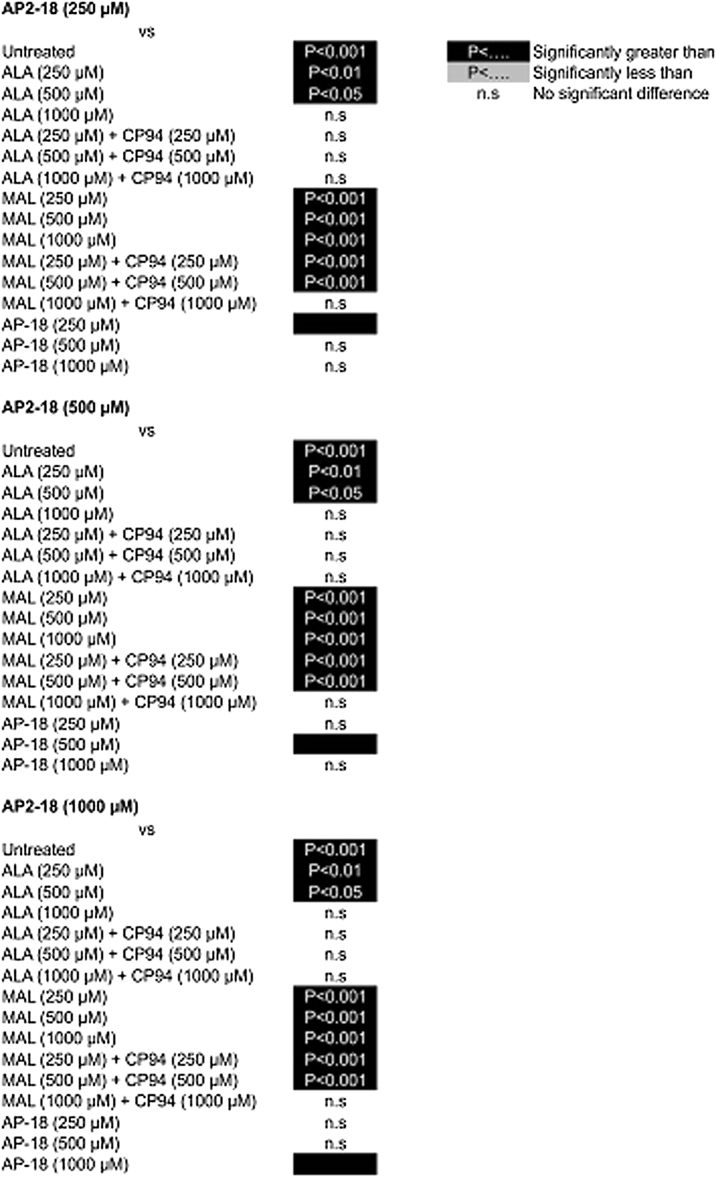


## References

[1] Lomas A., Leonardi-BeeJ J., Bath-Hextall F. (2012). A systematic review of worldwide incidence of nonmelanoma skin cancer. Br. J. Dermatol..

[2] Kim Y., He Y. (2014). Ultraviolet radiation-induced non-melanoma skin cancer: regulation of DNA damage repair and inflammation. Genes Dis..

[3] Vallejo-Torres L., Morris S., Kinge J., Poirier V., Verne J.J. (2014). Public Health.

[4] Taylor E.L., Brown S.B. (2002). The advantages of aminolevulinic acid photodynamic therapy in dermatology. J. Dermatol. Treat..

[5] Steinbauer J.M., Schreml S., Kohl E.A., Karrer S., Landthaler M., Szeimies R.-M. (2010). Photodynamic therapy in dermatology. JDDG: Journal der Deutschen Dermatologischen Gesellschaft.

[6] O’Connor A.E., Gallagher W.M., Byrne A.T. (2009). Porphyrin and nonporphyrin photosensitizers in oncology: preclinical and clinical advances in photodynamic therapy. Photochem. Photobiol..

[7] Choudhary S., Nouri K., Elsaie M.L. (2009). Photodynamic therapy in dermatology: a review. Lasers Med. Sci..

[8] Morton C., Szeimies R., Sidoroff A., Braathen L. (2013). European guidelines for topical photodynamic therapy part 1: treatment delivery and current indications - actinic keratoses, bowen’s disease, basal cell carcinoma. JEADV.

[9] Morton C.A., McKenna K.E., Rhodes L.E. (2008). Guidelines for topical photodynamic therapy: update. Br. J. Dermatol..

[10] Juarranz Á, Jaén P., Sanz-Rodríguez F., Cuevas J., González S. (2008). Photodynamic therapy of cancer. Basic principles and applications. Clin. Translat. Oncol..

[11] Pye A., Campbell S., Curnow A. (2008). Enhancement of methyl-aminolevulinate photodynamic therapy by iron chelation with CP94: an in vitro investigation and clinical dose-escalating safety study for the treatment of nodular basal cell carcinoma. J. Cancer Res. Clin. Oncol..

[12] Bown S.G. (2012). How mainstream medicine sees photodynamic therapy in the United Kingdom. J. Natl. Comprehens. Cancer Netw..

[13] Fayter D., Corbett M., Heirs M., Fox D., Eastwood A. (2010). A systematic review of photodynamic therapy in the treatment of pre-cancerous skin conditions, Barrett’s oesphagus and cancers of the biliary tract, brain, head and neck, lung, oesophagus and skin. Health Technol. Assess..

[14] Babilas P., Schreml S., Landthaler M., Szeimies R. (2010). Photodynamic therapy in dermatology: state-of-the-art. Photoderm. Photoimmun. Photomed..

[15] Sakamoto F.H., Lopes J.D., Anderson R.R. (2010). Photodynamic therapy for acne vulgaris: a critical review from basics to clinical practice: part I. Acne vulgaris: when and why consider photodynamic therapy?. J. Am. Acad. Dermatol..

[16] Peng Q., Berg K., Moan J., Kongshaug M., Nesland J. (1997). 5-aminolevulinic acid-based photodynamic therapy: principles and experimental research. Photochem. Photobiol..

[17] Peng Q., Warloe T., Berg K., Moan J., Kongshaug M., Giercksky K.E., Nesland J.M. (1997). 5-aminolevulinic acid-based photodynamic therapy. Clinical research and future challenges. Cancer.

[18] Kennedy J.C., Pottier R.H. (1992). Endogenous protoporphyrin IX, a clinically useful photosensitizer for photodynamic therapy. J. Photochem. Photobiol. B: Biol..

[19] Lang K., Bolsen K., Stahl W., Ruzicka T., Sies H., Lehmann P. (2001). The 5-aminolevulinic acid-induced porphyrin biosynthesis in benign and malignant cells of the skin. J. Photochem. Photobiol. B: Biol..

[20] Luna M.C., Ferrario A., Wong S., Fisher A.M., Gomer C.J. (2000). Photodynamic therapy-mediated oxidative stress as a molecular switch for the temporal expression of genes ligated to the human heat shock promoter. Cancer Res..

[21] Dirschka T., Radny P., Dominicus R., Mensing H., Brüning H., Jenne L. (2012). Photodynamic therapy with BF-200 ALA for the treatment of actinic keratosis: results of a multicentre, randomized, observer-blind phase III study in comparison with a registered methyl-5-aminolaevulinate cream and placebo. Br. J. Dermatol..

[22] Tyrrell J., Morton C., Campbell S., Curnow A. (2011). Comparison of PpIX accumulation and destruction during methyl-aminolevulinate photodynamic therapy (MAL-PDT) of skin tumours located at acral and non-acral sites. Br. J. Dermatol..

[23] Annemans L., Caekelbergh K., Roelandts R., Boonen H., Leys C., Nikkels A.F., van Den Haute V., van Quickenborne L., Verhaeghe E., Leroy B. (2008). Real-life practice study of the clinical outcome and cost-effectiveness of photodynamic therapy using methyl aminolevulinate (MAL-PDT) in the management of actinic keratosis and basal cell carcinoma. Euro J. Derm..

[24] Basset-Seguin N., Ibbotson S.H., Emtestam L., Tarstedt M., Morton C., Maroti M., Calzavara-Pinton P., Varma S., Roelandts R., Wolf P. (2008). Topical methyl aminolaevulinate photodynamic therapy versus cryotherapy for superficial basal cell carcinoma: a 5 year randomized trial. Euro J. Derm..

[25] Kaufmann R., Spelman L., Weightman W., Reifenberger J., Szeimies R.M., Verhaeghe E., Kerrouche N., Sorba V., Villemagne H., Rhodes L.E. (2008). Multicentre intraindividual randomized trial of topical methyl aminolaevulinate-photodynamic therapy vs. cryotherapy for multiple actinic keratoses on the extremities. Br. J. Dermatol..

[26] Morton C.A., Horn M., Leman J., Tack B., Bedane C., Tijoe M., Ibbotson S., Khemis A., Wolf P. (2006). Comparison of topical methyl aminolevulinate photodynamic therapy with cryotherapy or fluorouracil for treatment of squamous cell carcinoma in situ: results of a multicenter randomized trial. Arch. Derm..

[27] Szeimies R., Ibbotson S., Murrell D., Rubel D., Frambach Y., de Berker D. (2008). A clinical study comparing methyl aminolevulinate photodynamic therapy and surgery in small superficial basal cell carcinoma (8-20 mm), with a 12-month follow-up. JEADV.

[28] Hanania J., Malik Z. (1992). The effect of EDTA and serum on endogenous porphyrin accumulation and photodynamic sensitization of human K562 leukemic cells. Cancer Lett..

[29] Liu H.F., Xu S.Z., Zhang C.R. (2004). Influence of CaNa2 EDTA on topical 5-aminolaevulinic acid photodynamic therapy. Chin. Med. J. (Engl.).

[30] Malik Z., Kostenich G., Roitman L., Ehrenberg B., Orenstein A. (1995). Topical application of 5-aminolevulinic acid, DMSO and EDTA: protoporphyrin IX accumulation in skin and tumours of mice. J. Photochem. Photobiol. B: Biol..

[31] Orenstein A., Kostenich G., Roltman L., Shechtman Y., Kopolovic Y., Ehrenberg B., Malik Z. (1996). A comparative study of tissue distribution and photodynamic therapy selectivity of chlorin e6, photofrin II and ALA-induced protoporphyrin IX in a colon carcinoma model. Br. J. Cancer.

[32] Messmann H., Mlkvy P., Buonaccorsi G., Davies C.L., MacRobert A.J., Bown S.G. (1995). Enhancement of photodynamic therapy with 5-aminolaevulinic acid-induced porphyrin photosensitisation in normal rat colon by threshold and light fractionation studies. Br. J. Cancer.

[33] Curnow A., McIlroy B.W., Postle-Hacon M.J., MacRobert A.J., Bown S.G. (1999). Light dose fractionation to enhance photodynamic therapy using 5-aminolevulinic acid in the normal rat colon. Photochem. Photobiol..

[34] Robinson D.J., de Bruijn H.S., van der Veen N., Stringer M.R., Brown S.B., Star W.M. (1998). Fluorescence photobleaching of ALA-induced protoporphyrin IX during photodynamic therapy of normal hairless mouse skin: the effect of light dose and irradiance and the resulting biological effect. Photochem. Photobiol..

[35] Peng Q., Warloe T., Moan J., Godal A., Apricena F., Giercksky K.E., Nesland J.M. (2001). Antitumor effect of 5-aminolevulinic acid-mediated photodynamic therapy can be enhanced by the use of a low dose of photofrin in human tumor xenografts. Cancer Res..

[36] Juzeniene A., Juzenas P., Bronshtein I., Vorobey A., Moan J. (2006). The influence of temperature on photodynamic cell killing in vitro with 5-aminolevulinic acid. J. Photochem. Photobiol. B: Biol..

[37] Orenstein A., Kostenich G., Kopolovic Y., Babushkina T., Malik Z. (1999). Enhancement of ALA-PDT damage by IR-induced hyperthermia on a colon carcinoma model. Photochem. Photobiol..

[38] Lopez R.F., Bentley M.V., Delgado-Charro M.B., Salomon D., van den Bergh H., Lange N., Guy R.H. (2003). Enhanced delivery of 5-aminolevulinic acid esters by iontophoresis in vitro. Photochem. Photobiol..

[39] Bremner J.C., Adams G.E., Pearson J.K., Sansom J.M., Stratford I.J., Bedwell J., Bown S.G., MacRobert A.J., Phillips D. (1992). Increasing the effect of photodynamic therapy on the RIF-1 murine sarcoma, using the bioreductive drugs RSU1069 and RB6145. BJC.

[40] Berg K., Anholt H., Bech O., Moan J. (1996). The influence of iron chelators on the accumulation of protoporphyrin IX in 5-aminolaevulinic acid-treated cells. Br. J. Cancer.

[41] May B., Bhasker C., Bawden M., Cox T. (1990). Molecular regulation of 5-aminolevulinate synthase. Diseases related to heme biosynthesis. Mol. Biol. Med..

[42] Pye A., Curnow A. (2007). Direct comparison of delta-aminolevulinic acid and methyl-aminolevulinate-derived protoporphyrin IX accumulations potentiated by desferrioxamine or the novel hydroxypyridinone iron chelator CP94 in cultured human cells. Photochem. Photobiol..

[43] Curnow A., Pye A. (2007). Biochemical manipulation via iron chelation to enhance porphyrin production from porphyrin precursors. J. Environ. Pathol. Toxicol. Oncol..

[44] Blake E., Allen J., Curnow A. (2011). An in vitro comparison of the effects of the iron-chelating agents, CP94 and dexrazoxane, on protoporphyrin IX accumulation for photodynamic therapy and/or fluorescence guided resection. Photochem. Photobiol..

[45] Bech O., Phillips D., Moan J., MacRobert A. (1997). A hydroxypyridinone (CP94) enhances protoporphyrin IX formation in 5-aminolaevulinic acid treated cells. J. Photochem. Photobiol. B.

[46] Curnow A., McIlroy B., Postle-Hacon M., Porter J., MacRobert A., Bown S. (1998). Enhancement of 5-aminolaevulinic acid-induced photodynamic therapy in normal rat colon using hydroxypyridinone iron-chelating agents. Br. J. Cancer.

[47] Curnow A., MacRobert A., Bown S. (2006). Comparing and combining light dose fractionation and iron chelation to enhance experimental photodynamic therapy with aminolevulinic acid. Lasers Surg. Med..

[48] Curnow A., MacRobert A., Bown S. (2015). Enhancing protoporphyrin IX-induced photodynamic therapy with a topical iron chelating agent in a Normal skin model. J. Heavy Metal Chelation Therapy.

[49] Chang S., MacRobert A., Porter J., Bown S. (1997). The efficacy of an iron chelator (CP94) in increasing cellular protoporphyrin IX following intravesical 5-aminolaevulinic acid administration: an in vivo study. J. Photochem. Photobiol. B.

[50] Connell R., Curnow A., Cutner A., Bown S. (2000). Endometrial ablation in the rabbit uterus by photodynamic therapy (PDT) using 5-aminolaevulinic acid with the iron chelator CP94. Br. J. Obst. Gynaecol..

[51] Porter J., Morgan J., Hoyes K., Burke L., Huehns E., Hider R. (1990). Relative oral efficacy and acute toxicity of hydroxypyridin-4-one iron chelators in mice. Blood.

[52] Porter J., Hoyes K., Abeysinghe R., Brooks P., Huehns E., Hider R. (1991). Comparison of the subacute toxicity and efficacy of 3-hydroxypyridin-4-one iron chelators in overloaded and nonoverloaded mice. Blood.

[53] Bergeron R., Streiff R., Wiegand J., Luchetta G., Creary E., Peter H. (1992). A comparison of the iron-clearing properties of 1,2-dimethyl-3-hydroxypyrid-4-one, 1,2-diethyl-3-hydroxypyrid-4-one, and deferoxamine. Blood.

[54] Porter J., Abeysinghe R., Hoyes K., Barra C., Huehns E., Brooks P., Blackwell M., Araneta M., Brittenham G., Singh S., Bobbin P., Hider R. (1993). Contrasting interspecies efficacy and toxicology of 1,2-diethyl-3-hydroxypyridin-4-one, CP94, relates to differing metabolism of the iron chelating site. Br. J. Haematol..

[55] Fredenburg A., Wedlund P., Skinner T., Damani L., Hider R., Yokel R. (1993). Pharmacokinetics of representative 3-hydroxypyridin-4-ones in rabbits: CP20 and CP94. Drug Metab. Dispos..

[56] Campbell S., Morton C., Alyahya R., Horton S., Pye A., Curnow A. (2008). Clinical investigation of the novel iron-chelating agent, CP94, to enhance topical photodynamic therapy of nodular basal cell carcinoma. Br. J. Dermatol..

[57] Epemolu R., Ackerman R., Porter J., Hider R., Damani L., Singh S. (1994). HPLC determination of 1,2-diethyl-3-hydroxypyridin-4-one (CP94), its iron complex [Fe(III) (CP94)3] and glucuronide conjugate [CP94-GLUC] in serum and urine of thalassaemic patients. J. Pharm. Biomed. Anal..

[58] Kontoghiorghes G., Bartlett A., Hoffbrand A., Goddard J., Sheppard L., Barr J., Nortey P. (1990). Long-term trial with the oral iron chelator 1,2-dimethyl-3-hydroxypyrid-4-one (L1). I. Iron chelation and metabolic studies. Br. J. Haematol..

[59] Kersten M., Lange R., Smeets M., Vreugdenhil G., Roozendaal K., Lameijer W., Goudsmit R. (1996). Long-term treatment of transfusional iron overload with the oral iron chelator deferiprone (L1): a Dutch multicenter trial. Ann. Hematol..

[60] Kontoghiorghes G., Goddard J., Bartlett A., Sheppard L. (1990). Pharmacokinetic studies in humans with the oral iron chelator 1,2-dimethyl-3-hydroxypyrid-4-one. Clin. Pharmacol. Ther..

[61] Brittenham G. (1992). Development of iron-chelating agents for clinical use. Blood.

[62] Mobarra N., Shanaki M., Ehteram H., Nasiri H., Sahmani M., Saeidi M., Goudarzi M., Pourkarim H., Azad M. (2016). A review on iron chelators in treatment of iron overload syndromes. Int. J. Hematol. Oncol. Stem Cell Res..

[63] Bollig C., Schell L., Rücker G., Allert R., Motschall E., Niemeyer C., Bassler D., Meerpohl J. (2017). Deferasirox for managing iron overload in people with thalassaemia. Cochrane Database Syst. Rev..

[64] Curnow A, Wood M, Perry A. Pyridinone compounds for use in photodynamic therapy. WO2014033477A1, 06/03/14.

[65] Fautz R., Husein B., Hechenberger C. (1991). Application of the neutral red assay (NR assay) to monolayer cultures of primary hepatocytes: Rapid colorimetric viability determination for the unscheduled DNA synthesis test (UDS). Mutat. Res..

[66] Papini E., de Bernard M., Milia E., Bugnoli M., Zerial M., Rappuoli R., Montecucco C. (1994). Cellular vacuoles induced by helicobacter pylori originate from late endosomal compartments. PNAS.

[67] Blake E., Curnow A. (2010). The hydroxypyridinone iron chelator CP94 can enhance PpIX-induced PDT of cultured human glioma cells. Photochem. Photobiol..

[68] Maisch T., Santarelli F., Schreml S., Babilas P., Szeimies R.M. (2010). Fluorescence induction of protoporphyrin IX by a new 5-aminolevulinic acid nanoemulsion used for photodynamic therapy in a full-thickness ex vivo skin model. Exp. Dermatol..

[69] Curnow A., Pye A. (2015). The importance of iron chelation and iron availability during PpIX-induced photodynamic therapy. Photon. Lasers Med..

[70] Anayo L., Magnussen A., Perry A., Wood M., Curnow A. (2018). An experimental investigation of a novel iron chelating protoporphyrin IX prodrug for the enhancement of photodynamic therapy. Lasers Surg. Med..

[71] Schulten R., Novak B., Schmitz B., Lübbert H. (2012). Comparison of the uptake of 5-aminolevulinic acid and its methyl ester in keratinocytes and skin. Naunyn. Schmiedebergs Arch. Pharmacol..

[72] Battah S., Hider R., MacRobert A., Dobbin P., Zhou T. (2017). Hydroxypyridinone and 5-aminolaevulinic acid conjugates for photodynamic therapy. J. Med. Chem..

[73] Donnelly R., McCarron P., Woolfson A. (2007). Derivatives of 5-aminolevulinic acid for photodynamic therapy. Perspect. Med. Chem..

