# Supplementary material for: Improving in vitro photodynamic therapy through the development of a novel iron chelating aminolaevulinic acid prodrug
Source: Photodiagnosis Photodyn Ther. 2019 Mar;25:157–65. doi: 10.1016/j.pdpdt.2018.12.005 (PMC6456724; doi:10.1016/j.pdpdt.2018.12.005)
Supplement: Supplementary file 1 [file mmc1.docx]

**Supplementary Figure Legends**

**S1.** Statistical comparison (2 way ANOVA with Bonferroni post-test to compare replicate means) of PpIX fluorescence accumulation over time of the three concentrations of each compound tested (AP2-18, ALA alone, ALA + the iron chelator CP94, MAL alone and MAL + the iron chelator CP94) in human dermal fibroblasts (84BR).

**S2.** Statistical comparison (2 way ANOVA with Bonferroni post-test to compare replicate means) of PpIX fluorescence accumulation over time of the three concentrations of each compound tested (AP2-18, ALA alone, ALA + the iron chelator CP94, MAL alone and MAL + the iron chelator CP94) in human epithelial squamous carcinoma cells (A431).

**S3.** Statistical comparison (1 way ANOVA with Tukey post-test comparing all pairs of columns) of the PDT efficacy produced by the three concentrations of each compound tested (AP2-18, ALA alone, ALA + the iron chelator CP94, MAL alone and MAL + the iron chelator CP94) on irradiation with red light in human dermal fibroblasts (84BR).

**S4.** Statistical comparison (1 way ANOVA with Tukey post-test comparing all pairs of columns) of the PDT efficacy produced by the three concentrations of each compound tested (AP2-18, ALA alone, ALA + the iron chelator CP94, MAL alone and MAL + the iron chelator CP94) on irradiation with red light in human epithelial squamous carcinoma cells (A431).
